# Polymeric biomaterials in the treatment of posterior segment diseases

**DOI:** 10.3389/fmed.2022.949543

**Published:** 2022-08-18

**Authors:** Ivan Seah, Charles Ong, Zengping Liu, Xinyi Su

**Affiliations:** ^1^Department of Ophthalmology, National University Hospital, Singapore, Singapore; ^2^Department of Ophthalmology, Yong Loo Lin School of Medicine, National University of Singapore, Singapore, Singapore; ^3^Institute of Molecular and Cell Biology (IMCB), Agency for Science, Technology and Research (A^*^STAR), Singapore, Singapore; ^4^Singapore National Eye Centre (SNEC), Singapore, Singapore; ^5^Singapore Eye Research Institute (SERI), Singapore, Singapore

**Keywords:** retinal detachments, drug delivery system, macular degenerations, polymer, stem cell, retinal disease

## Abstract

Polymeric biomaterials are biological or synthetic substances which can be engineered to interact with biological systems for the diagnosis or treatment of diseases. These biomaterials have immense potential for treating eyes diseases, particularly the retina—a site of many inherited and acquired diseases. Polymeric biomaterials can be engineered to function both as an endotamponade agent and to prevent intraocular scarring in retinal detachment repair surgeries. They can also be designed as a drug delivery platform for treatment of retinal diseases. Finally, they can be used as scaffolds for cellular products and provide non-viral gene delivery solutions to the retina. This perspective article explains the role of polymeric biomaterials in the treatment of retinal conditions by highlighting recent advances being translated to clinical practice. The article will also identify potential hurdles to clinical translation as future research directions in the field.

## History of biomaterials in ophthalmology

Biomaterials are engineered substances which can interact with biological systems to diagnose or treat diseases. Early biomaterials were designed to perform mainly mechanical functions as implants. These implants can be found in all specialties of medicine. For example, metal and alloys like titanium are commonly used to produce heart valves, vessel stents and joint implants due to their high mechanical strength, immunity to corrosion and complete inertness to the body environment (1). Meanwhile bio-ceramics such as hydroxyapatite are used as bone and dental implants due to their excellent biocompatibility and wear resistance (2).

Unlike other body systems, the eye confers several key advantages for biomaterial applications. Firstly, the immune privilege status of the eye allows foreign biomaterials to be introduced with limited immunogenicity (3). Secondly, the existence of the blood-retinal-barrier limits the systemic penetration of most biomaterials. Furthermore, the small volume of biomaterial administered in the eye is unlikely to cause severe systemic effects. In addition, the implanted materials can be non-invasively monitored by state-of-art multi-modal ophthalmic investigations. Thus, the eye is an extremely attractive organ for application of novel biomaterials.

The earliest applications of biomaterials in the eye were intraocular implants for cataract replacement (4). Cataract is the most common reversible cause of visual loss affecting approximately 16 million people globally (5). Cataract surgery involves removing the opacified lens and implanting an artificial intraocular lens (IOL). The earliest lenses developed were made of polymethylmethacrylate due to its inert nature and clarity (6). However, with the advent of smaller surgical incisions and phacoemulsification cataract surgery, silicone and acrylic IOLs are widely used today as they have mechanical flexibility, allowing them to be folded for insertion through small incisions (7).

Glaucoma drainage device, like the Ahmed tube, are examples of more recent implants used for the treatment of advanced glaucoma. To achieve optimal intraocular pressure, the tube-shaped glaucoma drainage device, acts as a conduit to shunt aqueous humor away from the anterior chamber of the eye (8). These devices are usually made of silicone and polypropylene due to their flexibility and inertness. Separately, silicone is also used to manufacture scleral buckle implants, used in the surgical treatment of retinal detachments. In orbital reconstructive surgery for fractures, hydroxyapatite and porous polyethylene are used as orbital implants post enucleation (9).

## Polymeric biomaterials and posterior segment applications

Advances in material science have enabled the development of next generation biomaterials through modifications at the molecular level. Polymeric biomaterials are one such example. They are made of repeated subunits of smaller molecules, and can be naturally-derived or synthetic. Naturally-derived polymers can be made of polysaccharides, polypeptides or polynucleotides. These can be further modified chemically to confer useful properties (10). Synthetic polymers are man-made and generated either through step-growth polymerization or chain-polymerization. Step-growth polymerization involves the addition of a single monomer per reaction while chain-polymerization allows the addition of another polymer chain to the original polymer. These processes have allowed the creation of novel polymeric biomaterials with unique properties for treating eye diseases. A key example is sodium hyaluronate, which has been widely adopted as viscoelastic agents for use during cataract surgery (11).

The posterior segment of the eye consists of key structures like the vitreous humor, retina, choroid and optic nerve. These structures are affected in diseases such as retinal detachment, proliferative vitreoretinopathy, retinal neovascular diseases and inherited retinal diseases. For the posterior segment of the eye, various polymeric biomaterials are currently being developed as vitreous substitutes, sustained and topical drug delivery systems, scaffolds for cellular therapeutics and non-viral gene delivery agents (Table 1). These polymers will be discussed in greater detail in the next segment.

**Table 1:** Examples of biomaterials and applications in the posterior segment of the eye.

**Polymeric biomaterial**	**Therapeutic area**	**Application**	**Properties of biomaterial suitable for application**
EPC Thermogel - Polyethylene glycol, poly(propylene glycol), poly(e-caprolactone) (12, 13)	Retinal Detachment	• Retinal tamponade agent	• Optical clarity • Low swelling counter force • Biodegradable • Biocompatible • Able to maintain prolonged endotamponade effect • Able to suppress development of proliferative vitreoretinopathy • Able to regenerate vitreous-like material over time
EPC Nanomicelles - Polyethylene glycol, poly(propylene glycol), poly(e-caprolactone) (14)	Neovascular Retinal Diseases • Age-related macular degeneration • Diabetic retinopathy and macular oedema	Drug delivery agent	• Enables topical delivery of drugs to the posterior segment of the eye • Enables delivery of biologics such as FDA-approved anti-vascular endothelial growth factor compounds • Retains bioactivity of delivered drug to reduce area of neovascularisation • Biocompatible
Polyethylenimine (PEI)-based polymers (15, 16)	Inherited retinal diseases	Gene delivery agent	• Able to form DNA-polymer complexes • Enables delivery of genetic cargo into the cell of interest • Able to undergo endosomal release to release genetic cargo intracellularly • Biocompatible
Polyethylene terephthalate (PET) (17) Poly(lactic-co-glycolic acid) (PLGA) (18)	Advanced retinal degeneration • Inherited retinal diseases • Age-related macular degeneration	Scaffold for cellular therapeutics	• Biocompatible • Biodegradable • Able to promote and maintain a long-term retinal cell (RPE/photoreceptor) phenotype • Have durability to tolerate implantation procedure • Able to mimic physiological properties of retinal tissue

### Polymeric biomaterials in the treatment of retinal detachments and proliferative vitreoretinopathy

Retinal detachment is characterized by the separation of the neuroretina from the underlying retinal pigment epithelium. In vitreo-retinal surgery, after repairing the detachment and closing the retinal break, the vitreous cavity is replaced with an endotamponade agent to keep the retina attached during post-operative recovery. Traditional endotamponade agents include intraocular gas agents, like sulfur hexafluoride (SF_6_) and perfluoropropane (C_3_F_8_), or non-biodegradable fluids such as silicone oil. Intraocular gases can result in corneal decompensation and cataract formation. Patients also have to adopt a prolonged face-down position. Most importantly, these patients cannot partake in air travel as low atmospheric pressure can result in gaseous expansion. Meanwhile, prolonged use of silicone oil can cause corneal decompensation, if migrated into the anterior chamber, and even silicone oil glaucoma (19). Thus, the use of silicone oil is usually accompanied by a second removal surgery, inadvertently predisposing a patient to further surgical risks.

Despite the shortfalls of both modalities, innovation in this field has largely been stagnant since the 1970s. Apart from biocompatibility, an endotamponade agent should ideally be: (1) optically clear to allow visual inspection of the retina during follow-ups, (2) easily administered during surgery, (3) exert sufficient surface tension across the retina to allow adequate tamponade of the repaired retina, and (4) biodegradable to avoid a removal surgery. Polymeric hydrogels have the potential to fulfill these criteria through careful polymer selection and various chemical modifications (20–25). A hydrogel is comprised of a three-dimensional network of hydrophilic polymers. Due to the cross-linkages between the individual polymer chains, the polymers can retain water molecules to maintain a gel-like property. In recent years, “smart” hydrogels which are capable of changing states from solution to gel in response to physical and chemical stimuli have also been developed (26). In particular, thermogels can change states based on the surrounding temperature (27, 28). Our group previously developed a urethane-based thermogel of a polyethylene glycol (PEG), poly(propylene glycol) (PPG) and poly(e-caprolactone) (PCL) polymer, termed EPC. The thermogel exists in liquid form at lower temperatures, and turns into gel status at body temperature. This unique feature allows easy administration by injecting *via* 25-gauge syringe into the vitreous cavity and enables the tamponade effect once heated to body temperature. While other polymeric hydrogel formulations have been researched as endotamponade agents (29), EPC's has distinct advantages of biodegradability and low swelling counter force. This allows patients to avoid a second removal surgery, with low risk of raised intraocular pressure. Interestingly, when we implanted EPC in vitrectomized rabbit eyes, we observed a vitreous-like substance re-formed after EPC biodegradation. Further characterization of the vitreous-like substance is currently underway to determine the possibility of vitreous regeneration associated with the polymer (12, 30).

5–10% of all retinal detachment surgeries fail due to proliferative vitreoretinopathy (PVR), a major complication characterized by the development of contractile cellular membranes, leading to tractional retinal detachments (31). Current treatment relies primarily on surgical removal of these membranes. We recently demonstrated that EPC hydrogel is able to function as a bio-functional polymer to prevent retinal scarring in an experimental rabbit model of proliferative vitreo-retinopathy *via* the nuclear factor erythroid 2-related factor (NRF2) signaling pathway. This is a first report, whereby a synthetic polymeric material alone can target intracellular signaling pathways to prevent retinal scarring. More importantly, it offers insight into how synthetic polymeric materials no longer function merely as inert drug carriers and challenges the conventional belief that a small molecule (drug) is always required to achieve a therapeutic effect at a cellular level. It lays the foundation for next generation nanomedicine, whereby polymers alone can be used to elicit specific biological responses (13).

### Polymeric biomaterials in the treatment of retinal neovascular diseases

Retinal diseases such as age-related macular degeneration (AMD), diabetic retinopathy (DR) and diabetic macular oedema (DMO) affect a significant amount of people globally. For instance, AMD is expected to affect approximately 288 million people by 2040 (32). Significant visual loss may occur in advanced disease. Due to the significant disease burden of retinal diseases, and dramatic reduction in quality of life due to vision loss, significant effort is being channeled into developing effective therapeutics. A notable breakthrough in the field was the development of intravitreal anti-vascular endothelial growth factor (anti-VEGF) treatment for neovascular retinal diseases in the early 2000s (33). Anti-VEGF therapies include bevacizumab (Avastin^®^) and FDA-approved, ranibizumab (Lucentis^®^) and aflibercept (Eylea^®^). These drugs have to be given by intravitreal injections at regular frequencies of between 1 to 3 months (34), which many patients find troublesome, resulting in reduced treatment compliance. Furthermore, intravitreal injections are associated with sight-threatening risks such as increased ocular pressure, retinal detachment, vitreous hemorrhage and endophthalmitis (35). The holy grail of drug delivery is to develop novel solutions for sustained delivery, thereby reducing the frequency of injections. One such example, is a poly lactide-co-glycolide (PLGA) implant that delivers dexamethasone (Ozurdex^®^) for up to 3-months. Other non-polymeric approaches have been utilized to reduce the frequency of intravitreal injections. These include FDA-approved Brolucizumab (Brolu^®^), a humanized monoclonal single-chain variable fragment which has a longer duration of effect than current anti-VEGF compounds, and the Port Delivery System^®^ (PDS), a surgically implanted drug reservoir which allows the slow administration of ranibizumab over time. However, Brolucizumab is potentially associated with higher rates of intraocular inflammation, such as retinal vasculitis (36), while PDS requires an implantation surgical procedure, might be associated with increased risks of sight-threatening complications (37).

A key challenge in developing polymeric hydrogels suitable for sustained anti-VEGF delivery, is the preservation of antibody bioactivity during both hydrogel formulation and biodegradation. For instance, most hydrogels require chemical cross-linking agents for gelation. This can inadvertently result in drug inactivation. Thermogels, on the other hand, rely on small changes in temperature to induce physical cross-linking for gelation to occur, thus enabling preservation of anti-VEGF bioactivity. Indeed, we demonstrated in our EPC thermogel system, that sustained release of bevacizumab or aflibercept is achieved over 40 days, a duration greater than standard current clinical treatment interval. Furthermore, aflibercept released from biodegradable EPC gel retained its bioactivity and was capable of inhibiting vascular leakage in a rabbit choroidal neovascularisation model (38), suggesting the additional utility of the thermogel as a sustained drug release platform in the posterior segment of the eye.

Nano-micelles are globular structures which comprise of an internal hydrophobic fatty acyl chain and external hydrophilic polar head. The discovery of a topically administered nano-micellar system to deliver biologics to the retina—is the holy grail in ocular therapeutics—as it can overcome the multiple sight-threatening complications associated with invasive intravitreal injections. However, multiple static and dynamic ocular barriers between the cornea and the retina, prevent drugs from attaining a therapeutic concentration at the retina sufficient for disease control (39–42). A nano-micelle formulation of the EPC polymer is capable of topically delivering aflibercept to the retina of laser-induced choroidal neovascularisation (CNV) murine models. A single drop of the compound achieved a drug concentration in the murine vitreous that was above the clinically significant concentration required to inhibit VEGF activity. Most importantly, EPC micelle alone seems to have intrinsic anti-angiogenic properties, which works synergistically with aflibercept to result in CNV regression in murine models (14). Further characterization of the pharmacokinetics of EPC nano-micelles is currently underway. Topical compounds, if successful, have the potential to reduce the clinical burden of long-term clinical visits required for invasive intravitreal therapy.

### Future perspective: Biomaterials in retinal gene therapy for inherited retinal degenerations (IRDs)

Treatment options for IRDs are limited. Most of these patients eventually become visually impaired, relying on visual aids and rehabilitation for daily activities. To date, only 1 gene therapy exists for a specific IRD. Voretigene neparvovec (Luxturna^®^) is an adeno-associated serotype 2-delivered gene therapy for patients with biallelic RPE-65 mediated IRDs (43). RPE-65 mutations are usually associated with leber congenital amaurosis or retinitis pigmentosa. Since the FDA approval of this drug, many other novel gene therapeutic candidates have emerged. A key hurdle to developing a successful gene therapeutic is the method of gene delivery. Gene delivery platforms can be broadly classified into viral and non-viral methods (44). Viral methods are prevalently utilized as the adeno-associated viral (AAV) vector has been proven to be safe and effective through the approval of voretigene neparvovec. However, viral vectors have inherent limitations. The AAV vector has a packaging capacity of approximately 4.7kb (45). Hence, it is challenging to deliver larger genes such as *ABCA4* and *EYS* which are implicated in common IRDs such as Stargardt disease and retinitis pigmentosa (46, 47). Dual and even triple AAV strategies have been proposed but have suffered from poor transfection efficiency (48). Other viruses with larger packaging capacities may not demonstrate the same tropism and safety profile as AAV.

Cationic polymers are effective non-viral methods of gene delivery. Polyethylenimine (PEI) has demonstrated effective transfection of various human cell lines through the proton-sponge effect (49). However, the use of PEI is limited by its biocompatibility. Attempted strategies to improve biocompatibility include branching of PEI and conjugation with other polymers. For instance, Kurosaki et al. demonstrated that cationic complexes of DNA/PEI, when coated with γ-polyglutamic acid or chondroitin sulfate, was capable of *in-vivo* gene delivery when administered intravitreally into eyes of mice (15). Natural polymers, which may have better biocompatibility profiles, have also been trialed as gene delivery vectors. Liposomes, which are vesicles of lipid with 1 or more phospholipid bilayers enclosing an aqueous core have also demonstrated the ability to deliver the RPE-65 gene into knock out mice (16). Lipid nanoparticles, which are also lipid nano-formulations, may not necessarily have phospholipid bilayers that liposomes have. Solid lipid nanoparticles, when conjugated with dextran have shown the ability to achieve a transfection efficiency of 50% in RPE cells (50). These successes highlight the potential of using polymeric biomaterials for non-viral gene delivery, especially since the use of viral vectors also incurs enormous manufacturing costs.

### Future perspective: Biomaterials in cellular therapy for retinal degenerations

Unfortunately, gene therapy is only viable for early stage IRDs. For patients who have undergone significant disease progression, retinal stem cell therapeutics hold greater promise. Since the proof-of-concept clinical trial by Schwartz et al. on embryonic stem cell (ES) derived retinal cell transplantation, research in this field has evolved tremendously (51). Many factors that contribute to transplantation success have been studied. These include cell sources, methods of delivery, degree of cellular maturation and the need for immunosuppression (52).

Currently, retinal cellular therapeutics can be classified into either RPE or photoreceptor transplantation. RPE is a cell monolayer sitting beneath the photoreceptors and above Bruch's membrane, thus there is a need to use either biostable or biodegradable scaffolds as a cell carrier to support long term function of the transplanted RPE cells. Two types of biostable scaffolds, polyethylene terephthalate (PET) (17) and parylene (53), have been successfully used for RPE cell monolayer transplants in clinical trials in AMD patients. A PLGA-based electrospun nanofibrous scaffolds with RPE patch were tested for safety and efficiency in Royal College of Surgeons (RCS) rats and pigs (18). One should be cautious in using biodegradable scaffolds in patients, as the by-products of biodegradation might be toxic to retinal cells. Moreover, bio-safety of scaffolds and its ability to support graft survival has been proven (54, 55), further studies are required to determine the scaffold's role in establishing visual recovery.

In photoreceptor transplantation, the goal is to prevent remaining healthy photoreceptors from undergoing further degeneration and eventual apoptosis (56). This can be achieved by transplanting photoreceptor cells of varying cellular maturity. Many studies have suggested that the orientation of transplanted photoreceptors and how they interface with host photoreceptor cells play a crucial role in post-transplant survival and integration (57). Thus, a polymeric substrate supporting a monolayer of transplanted photoreceptors can potentially achieve this, and promote inter-digitation between transplanted photoreceptor outer segments and endogenous RPE microvilli (58).

## Translational hurdles

Polymeric biomaterials hold great potential for treating many retinal diseases. However, significant hurdles have to be overcome to fully achieve clinical translation.

Firstly, better characterization of polymer-tissue relationship is required. Polymer development is largely based on an iterative process which requires significant resources, time and intuition. Increasingly, the field is developing machine-learning tools to predict properties of polymers in-silico (59). This data-driven approach may enable rational polymer design based on the required function in the tissue type the polymer interacts with.

Secondly, the ocular pharmacokinetics of novel polymers have to be explored. Important aspects such as bio-distribution, metabolism and excretion mechanisms can affect the biocompatibility of the polymer significantly. While the systemic pharmacokinetics of polymers like PEG and PCL may have been established previously, their effects on local ocular tissue structure and function still needs to be studied in order to better understand the safety and clearance path of the byproducts while polymers are degrading (54).

Thirdly, manufacturing considerations have to be addressed for successful translation. A large part of the translation process is identifying a manufacturing process that is scalable, maintains the material's original physico-chemical properties and meets the sterility requirements of regulatory agencies. Most often, these aspects are inter-related. For instance, adjusting a polymer's structure to increase the mechanical strength can lead to the reduction of the material's clarity. Furthermore, common sterilization methods that are recognized by regulatory authorities are often physico-chemical methods involving heat, radiation and chemicals such as ethylene oxide, which may inadvertently alter the physico-chemical properties of polymers, rendering them unsuitable. Moreover, due to the complexity of the polymer synthesis processes, adequate monitoring and quality control is required to ensure functionality of final polymer product. As such, the final polymer synthesis process optimized for industry manufacturing, may be very different from the initial conceptualized process laid out in the research laboratory.

## Conclusion

Biomaterials have played significant roles in ophthalmology including intraocular lenses and glaucoma drainage devices. The applications of biomaterials are rapidly expanding, especially for polymeric biomaterials. Today, many polymeric biomaterials are being studied to address the challenges of treating retinal diseases. These applications range from endotamponade agents in retinal detachment surgery, drug delivery for retinal neovascular diseases and gene/cell delivery carriers. However, many translational hurdles still exist. Future work should focus on understanding the ocular pharmacokinetics of novel polymeric biomaterials and establishing synthesis methods that are suitable for large-scale manufacturing.

## Author contributions

Project conceptualization: IS and XS. Data collection: IS and CO. Manuscript writing: IS, CO, ZL, and XS. Project supervision: XS. All authors contributed to the article and approved the submitted version.

## Funding

This work was supported by the National Research Foundation (NRF), Singapore, under its Competitive Research Programme (CRP) (NRF-CRP21-2018-00103), Agency for Science, Technology and Research (A^*^STAR), Singapore, and HMBS Domain under its Industry Alignment Fund Pre-Positioning (IAF-PP) programme (H20/H7/a0/034) awarded to XS.

## Conflict of interest

The authors declare that the research was conducted in the absence of any commercial or financial relationships that could be construed as a potential conflict of interest.

## Publisher's note

All claims expressed in this article are solely those of the authors and do not necessarily represent those of their affiliated organizations, or those of the publisher, the editors and the reviewers. Any product that may be evaluated in this article, or claim that may be made by its manufacturer, is not guaranteed or endorsed by the publisher.
